# Oral treatment with etoposide in small cell lung cancer – dilemmas and solutions

**DOI:** 10.2478/raon-2013-0008

**Published:** 2013-02-01

**Authors:** Renata Rezonja, Lea Knez, Tanja Cufer, Ales Mrhar

**Affiliations:** 1 Faculty of Pharmacy, University of Ljubljana, Ljubljana, Slovenia; 2 Krka, d.d., Novo mesto, Slovenia; 3 University Clinic Golnik, Golnik, Slovenia

**Keywords:** oral etoposide, bioavailability, pharmacokinetic variability, small cell lung cancer

## Abstract

**Background:**

Etoposide is a chemotherapeutic agent, widely used for the treatment of various malignancies, including small cell lung cancer (SCLC), an aggressive disease with poor prognosis. Oral etoposide administration exhibits advantages for the quality of life of the patient as well as economic benefits. However, widespread use of oral etoposide is limited by incomplete and variable bioavailability. Variability in bioavailability was observed both within and between patients. This suggests that some patients may experience suboptimal tumor cytotoxicity, whereas other patients may be at risk for excess toxicity.

**Conclusions:**

The article highlights dilemmas as well as solutions regarding oral treatment with etoposide by presenting and analyzing relevant literature data. Numerous studies have shown that bioavailability of etoposide is influenced by genetic, physiological and environmental factors. Several strategies were explored to improve bioavailability and to reduce pharmacokinetic variability of oral etoposide, including desired and undesired drug interactions (*e.g.* with ketoconazole), development of suitable drug delivery systems, use of more water-soluble prodrug of etoposide, and influence on gastric emptying. In addition to genotype-based dose administration, etoposide is suitable for pharmacokinetically guided dosing, which enables dose adjustments in individual patient.

Further, it is established that oral and intravenous schedules of etoposide in SCLC patients do not result in significant differences in treatment outcome, while results of toxicity are inconclusive. To conclude, the main message of the article is that better prediction of the pharmacokinetics of oral etoposide may encourage its wider use in routine clinical practice.

## Introduction

Etoposide is a topoisomerase II inhibiting anti-cancer drug, derived from podophyllotoxin. It has significant therapeutic activity in childhood leukemia, testicular tumors, Hodgkin’s disease, large cell lymphomas and small cell lung cancer (SCLC).^1^ In combined therapy with platinum compound (cisplatin or carboplatin), etoposide is used as a first-line therapy for SCLC, an aggressive disease with poor prognosis, which represents roughly 20% (15–25%) of all lung cancers.^2^–^7^ With etoposide and cisplatin or carboplatin combination an overall response rate of approximately 75% can be anticipated. Radiation therapy to the thorax in addition to platinum/etoposide chemotherapy is associated with a small, but significant improvement in local control and overall survival in limited-stage disease.^8^ Progress in the management of SCLC has been modest in recent years as initial results of cisplatinum plus irinotecan showed improved survival. However these results were not confirmed in subsequent trials and cisplatinum/etoposide chemotherapy remains cornerstone of treatment for patients with SCLC.^9^,^10^ In addition, some major contribution over the last 20 years has come also from radiotherapy.^11^

The mode of action of etoposide involves inhibition of topoisomerase II, a nuclear enzyme that is necessary for swivelling and relaxation of deoxyribonucleic acid (DNA) during replication and transcription. Etoposide inhibits the ability of topoisomerase II to relegate cleaved nucleic acid molecules by the formation and stabilisation of a topoisomerase II-etoposide-DNA ternary complex and thus increases topoisomerase II-mediated DNA breakage. The covalent topoisomerase II-cleaved DNA complex is normally a short-lived intermediate in the reaction and is tolerated by the cell. However, at high concentrations it has cytotoxic effects, probably due to impaired DNA repair, leading to apoptosis.^12^,^13^

The activity of etoposide is dose- and schedule-dependent, and etoposide efficacy might be improved markedly with repeated drug administration.^12^–^17^ Etoposide directly interacts with the ATP-bound enzyme monomer in such a way that each molecule of etoposide stabilizes only a single-stranded break. Depending on the dose of etoposide, single-stranded or double-stranded DNA breaks are generated.^13^,^14^ Furthermore, the inhibition of topoisomerase II by etoposide is reversible and discontinuation of ternary complex allows quick DNA repair and diminishes the cytotoxicity of the drug. Thus, prolonged exposure to etoposide could increase the anticancer activity of the drug.^12^ Moreover, topoisomerase II is significantly expressed only in dividing cells during S and G2 phases of the cell cycle. Chronic scheduling maximizes the likelihood of exposing malignant cells to etoposide during sensitive periods of the cell cycle.^14^ However, myelosupression as the dose-limiting toxicity should be taken into account when planning the chemotherapy regimen.^14^ The chemotherapy treatment is therefore given in cycles, attacking cancer cells at their most sensitive periods, and allowing normal body cells time to recover.

A wide range of doses and schedules of etoposide are in use, depending on the treated disease. In patients with solid tumors, including SCLC, lower doses, such as 50–100 mg/m^2^/day over 3–5 days are suggested by some authors, while other authors suggest prolonged schedule as superior.^18^–^20^ In most regimens etoposide is administered in cycles, which are usually repeated every 3–4 weeks.^1^,^21^

Etoposide is commercially available in both intravenous and oral formulations. The oral formulation exhibits advantages for the patient as well as economic benefit compared with the intravenous one. The work from Liu and coworkers has indicated that 89% of incurable cancer patients preferred oral over intravenous chemotherapy, predominantly because of the convenience of administration, problems with intravenous access or needles, and a better chemotherapy-taking environment (outside of the clinic).^22^ In general, the quality of life of patients receiving palliative chemotherapy for advanced cancer was significantly poorer in patients treated at hospital compared with those treated at home.^23^ Most importantly, the oral formulation may provide an attractive alternative for patients who are unable to or have difficulty making the necessary and frequent visits to receive intravenous therapy.^24^ In comparison with intravenous infusion, oral administration of etoposide represents a significant cost saving for hospital and health insurance. Results of an economic analysis, which was conducted within a randomised multicentre study comparing the use of intravenous etoposide versus oral etoposide treatment in SCLC patients, reported a 17% savings for patients receiving the oral regimen. The following costs were examined: antineoplastic drugs, intravenous fluids, supplies used for chemotherapy administration, and chemotherapy administration procedure fees.^25^ Furthermore, the introduction of oral etoposide into combination chemotherapy regimens may shorten the hospitalization period and thus reduce non-drug related treatment costs as well.^26^

However, despite the numerous advantages of oral therapy, the intravenous formulation has been used more extensively.^24^ The main drawback of oral etoposide is its incomplete and variable bio-availability.^27^ Approximately 50% (30–97%) of the oral dose is bioavailable when compared with the intravenous route.^26^–^29^ This means that the area under the curve (AUC) of a given oral dose is approximately 50% of what would be achieved after an intravenous dose. Additionally, variability in bioavailability was observed both within and between patients. Hande *et al.*^30^ reported a mean etoposide bioavailability at a dose of 50 mg 64.6%, with intrapatient coefficient of variation (CV) 22.6% and interpatient CV 34.8%. A large CV suggests that some patients are receiving inadequate drug exposure, resulting in suboptimal tumor cytotoxicity, whereas others may be at risk for excess toxicity.^30^ This is particularly important when using drugs with a narrow therapeutic window, like etoposide.^31^ Additionally, a linear pharmacokinetic behaviour of oral etoposide was shown only for doses up to 200 mg.^32^ In higher doses, the percentage of absorbed etoposide may decrease while the CV in oral etoposide bioavailability may even increase.^29^

The absorption of etoposide is likely to depend on a number of interacting factors, the identification of which may be difficult. The improvement in the absorption of etoposide and the reduction in its variability, remain important goals to facilitate the clinical use of oral etoposide.^27^

This review focuses on the impact of various factors influencing bioavailability of etoposide, provides possibilities for its improvement and suggestions for treatment optimisation to ensure comparable pharmacokinetic parameters of oral and intravenous application. The review is restricted to treatment with etoposide in SCLC patients, for which etoposide-platinum doublet still represents the most effective standard therapy.

## Bioavailability of oral etoposide

Bioavailability is the extent to which an administered drug enters the systemic circulation. It is defined by the AUC of the dose delivered by oral administration divided by the AUC of the intravenous application of the same dose. AUC of etoposide correlates with safety and efficacy as well as overall survival of patients with SCLC.^33^,^34^ Oral administration may increase AUC variability because the drug must undergo additional processes such as being transported across the intestine, passing through the liver, and entering the systemic plasma circulation.^30^ Those are pharmacokinetic processes called absorption, first-pass metabolism and elimination prior entering the systemic circulation.^35^ Variation in the pharmacokinetics of a drug in a patient population is the net result of many complex interactions between genetic, physiological and environmental factors.^36^ The impact of these factors on pharmacokinetic processes and consequently AUC and bioavailability is described in some details in the following sections.

### Genetic factors

Genetic characteristics of metabolizing enzymes and transporters may influence drug blood level. Inherited differences in enzymes and transporters are known examples of pharmacogenetic variability. These factors may lead to inter-individual variation.^37^

Etoposide is a substrate of the efflux membrane transporters (ABC transporters) and metabolizing enzymes, which are located in the intestine and liver. Efflux membrane transporters limit the absorption of orally administered drug in the intestine and facilitate the pre-systemic elimination via bile, leading to poor bioavailability of drugs.^38^ A study aiming to characterize the regional absorptive and secretory kinetics of etoposide in rabbit intestinal tissues revealed that the apical to basolateral (*i.e.* absorptive) transport of etoposide was not apparently mediated by specialized transporters, whereas basolateral to apical (*i.e.* secretory) transport by intestinal tissues was concentration dependent and saturable, mediated by transporters.^39^

**FIGURE 1.**
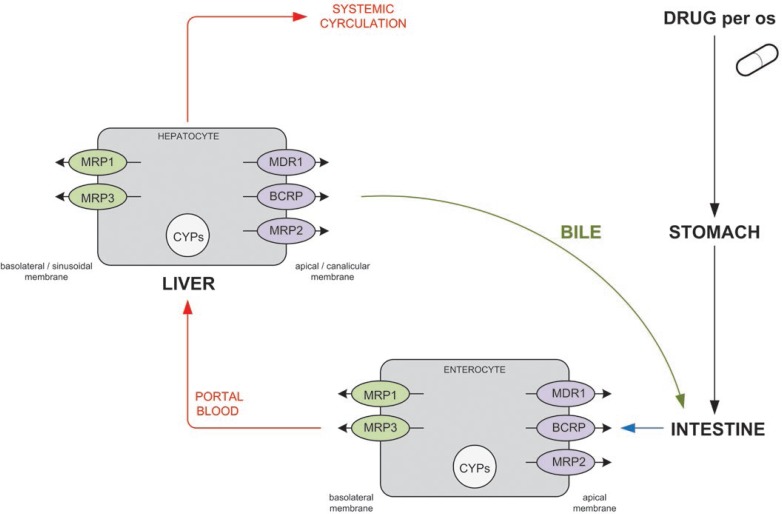
Schematic representation of the efflux transporters and metabolic enzymes (marked) possibly influencing etoposide bioavailability (modified by Ref. 58). MDR1 = multi-drug resistance protein (ABCB1, P-glycoprotein); MRP1-3 = multidrug resistance-associated proteins (ABCC1-3); BCRP = breast-cancer resistance protein (ABCG2); CYPs = cytochrome P450

Etoposide was shown to be a substrate of several ABC transporters, notably ABCB1 (MDR1, P-glycoprotein, P-gp) and ABCC1 (MRP1), ABCC2 (MRP2), ABCC3 (MRP3) and ABCG2 (BCRP).^40^–^45^ The location of these transporters in enterocytes and hepatocytes is marked in Figure 1. Allen *et al.*^40^ showed that ABCB1 can have a substantial effect on the oral availability of etoposide, while ABCG2 can have a little effect on oral etoposide pharmacokinetics. *In vitro* data showed that ABCC2 and ABCC3 can moderately transport etoposide.^42^,^43^ Etoposide was shown to be a good ABCC1 substrate.^44^ Lagas *et al.*^46^ studied the impact of ABCB1, ABCC2 and ABCC3 on the pharmacokinetics of etoposide in wild-type, ABCC2^−/−^, ABCB1a/1b^−/−^, and ABCB1a/1b;ABCC2^−/−^ mice. Results demonstrated that ABCB1, which is located in apical membrane of enterocytes, restricted the oral (re)uptake of unchanged etoposide, and mediated its excretion across the gut wall, while hepatobiliary excretion of both etoposide and etoposide glucuronide were almost entirely dependent on ABCC2, and not on ABCB1. Additionally, ABCC3 was responsible for the efflux of etoposide glucuronide from the liver to the systemic blood circulation, especially when ABCC2 was absent. Authors concluded that pharmacokinetics of etoposide and etoposide glucuronide is significantly affected by ABCB1, ABCC2, and ABCC3 and that high inter-individual variability of etoposide may be explained by variation in transporter expression or activity.^46^

Drug metabolism principally occurs in the liver, but also other tissues, like intestinal mucosa, must be considered.^12^ Etoposide is O-demethylated primarily by cytochrome P450 (CYP) 3A4 and to a lesser extent by CYP3A5.^47^,^48^ Furthermore, CYP1A2 and 2E1 are involved as the minor enzymatic components in this metabolic pathway.^49^ O-demethylated metabolite of etoposide is catehol.^47^ Catehol can undergo oxidation to form an ortho-quinone (and vice versa) via formation of a semi-quinone free radical. Studies suggest that radical species, in addition to the catehol and ortho-quinone, might also be involved in the cytotoxicity of etoposide.^50^–^52^ Ortho-quinone is attenuated by glutathione conjugation.^53^ The second way of etoposide metabolism is glucuronidation, mainly catalyzed by UGT1A1. Although etoposide glucuronidation is also catalyzed by UGT1A8 and 1A3, their activities are much lower than that of UGT1A1. The predominant form of etoposide glucuronide in liver and intestine is phenolic glucuronide, whereas two alcoholic glucuronides are the minor metabolites.^54^,^55^ CYP isoform was reported to be directly involved in the oxidative metabolism of etoposide, therefore variation of the intestinal activity of this CYP isoform may directly affect the bioavailability of etoposide.^12^

As shown in Figure 1, once etoposide as a drug crosses the apical membrane of the enterocyte, a part is effluxed back to the intestinal lumen by ABC transporters ABCB1 (MDR1), ABCC2 (MRP2) and ABCG2 (BCRP) and part is possibly subjected to intestinal first-pass metabolism by metabolizing enzymes. The fraction of drug absorbed into the mesenteric blood circulation enters into the liver via the portal vein and may be transported from hepatocytes into the bile (metabolized or non-metabolized) or to the systemic circulation.^56^,^57^

Many enzymes and secretory transporters are subject to genetic polymorphisms with functional consequences. A complete description of these polymorphisms can be found in the article of Robert *et al*.^59^ as well as on the dedicated websites (www.pharmgkb.org, www.imm.ki.se/CYPalleles, www.hapmap.org, www.ncbi.nlm.nih.gov/projects/SNP/). Genetic polymorphisms might be a one of the factors causing the interindividual differences in etoposide bioavailability. However, no study evaluated the association of these polymorphisms with etoposide bioavailability. Only one study explored the effect of polymorphisms in the *ABCB1* on etoposide pharmacokinetics. In this study *ABCB1* 3435TT genotype was associated with lower volume of distribution and contributed significantly to the inter-individual variability observed in etoposide pharmacokinetics.^60^ However, effects of *ABCB1* polymorphisms, particularly 3435C>T, on digoxin plasma levels after oral administration were extensively studied.^61^,^62^

Summarized, genetic variability and functional polymorphisms in ABC transporters are relevant pharmacological factors that have to be considered together with drug-metabolizing enzymes, whose activity show a large degree of interindividual variability.^37^

### Physiological factors

The metabolism of etoposide is partly hepatic, therefore hepatic insufficiency causes an increase in bioavailability of etoposide due to decreased first-pass effect.^12^,^63^ However, Hande *et al.*^29^,^30^ stated that variation in hepatic metabolism probably does not explain differences between oral and intravenous drug administration because etoposide’s hepatic clearance rate is not high. Aita *et al.*^64^ studied the pharmacokinetics of oral etoposide in patients with hepatocellular carcinoma and underlying cirrhosis. They found slightly high etoposide bioavailability and clearance resulting in a normal AUC.^64^ Bioavailability of etoposide was not affected neither in patients with gastric carcinoma nor in patients with gastrectomy.^65^

Ando *et al.*^66^ showed that gender does not affect the pharmacokinetics or pharmacodynamics of oral etoposide, while patient’s age affect pharmacodynamics. Although there was no difference in pharmacokinetics between elderly (ages 75 years or older) and younger patients, equivalent exposure to etoposide resulted in greater pharmacodynamic sensitivity in elderly patients.^66^,^67^ Contrary to the results of Ando *et al.*^66^, Miyazaki *et al.*^68^ showed that, although there were no significant differences in mean AUC values, plasma clearance and urinary excretion of oral etoposide, there were significant differences in elimination half life and bioavailability in the elderly group, compared with the younger adult group; both were significantly increased in the elderly patients. In comparison with intravenous administration, there was no statistically significant difference in these parameters between the elderly and younger adult group.^68^

### Environmental factors

Cancer patients commonly receive multiple medications, including chemotherapy and supportive care drugs, the majority of them are elderly, and so require medications for co-morbid conditions, and have age-related decline in hepatic and renal function that reduce their ability to metabolize and eliminate drugs.^69^ The possibility of drug-drug and drug-food interactions is therefore high. Interactions that affect bioavailability are usually pharmacokinetic interactions involving metabolising enzymes and drug transporters.

Many clinical and preclinical studies are documented wherein CYP450 and/or ABCB1 and/or UGT1A1 were prominently implicated to play an important role in etoposide bioavailability. Several CYP3A4 and ABCB1 inhibitors were described to enhance etoposide bioavailability, such as platinum compounds, cyclosporine A, hydroxyzine, quinidine, 20(S)-ginsenoside Rh2, GF120918, kaempferol, morin, quercetin, verapamil, PSC833 (valspodar), ketoconazole, piperine analogue and curcumin.

A study exploring the potential interaction between the two platinum drugs, cisplatin and carboplatin, and the oxidative metabolism of etoposide demonstrated that the interaction between etoposide and platinum drugs is small and the clinical impact is unlikely to be significant. The exact mechanism of interaction is unknown but may involve inhibition of etoposide metabolism.^70^

Cyclosporine A, hydroxyzine and quinidine were shown to increase systemic etoposide exposure through inhibition of the multidrug transporter ABCB1.^71^–^73^ Increased AUC and additionally peak concentration (c_max_) was observed by co-administration of an ABCB1 inhibitor 20(S)-ginsenoside Rh2, a trace constituent of ginseng.^74^ Increased oral uptake of etoposide due to ABCB1 inhibition was shown also by GF120918.^40^

The oral bioavailability of etoposide increased significantly when the drug was combined with kaempferol, morin or quercetin, three ingredients of dietary supplements. Additionally, kaempferol also increased c_max_ of oral etoposide. A possible explanation to enhanced bioavailability of oral etoposide by these three aforementioned drugs could be due to an inhibition of CYP450-catalyzed metabolism and ABCB1-mediated efflux in the intestine and/or liver.^75^–^77^ Similar results were obtained with verapamil and PSC833 (valspodar), a CYP3A and ABCB1 inhibitor.^40^,^78^,^79^

Ketoconazole was also shown to increase systemic exposure of oral etoposide. Ketoconazole is a commonly used antifugal drug known for its inhibitory effect on CYP3A4, UGT1A1, and ABCB1. However, Peng Yong *et al.*^80^ reported that increased systemic exposure to etoposide by ketoconazole modulation is most likely mediated through the inhibition of etoposide metabolism in the liver rather than the inhibition of the transporters in the intestine.^80^

The results of a study conducted by Harvey *et al.*^81^ showed that food does not significantly interfere with etoposide bioavailability, at least at doses of 100 mg. Grapefruit juice increases the bioavailability of some orally-administered drugs that are metabolized by CYP3A4. However, Reif *et al.*^82^ reported that coadministration of grapefruit juice causes an unexpected decrease in systemic exposure of oral etoposide. A possible explanation for the observed effect may be an alteration of the intestinal ABCB1-mediated transport.^82^ It was also shown that piperine analogue, a natural alkaloid of peppers significantly enhanced the plasma levels of etoposide. A mechanistic evaluation of this effect presented by Najar *et al.*^83^ has shown that piperine analogue modifies ABCB1 and CYP3A4-mediated drug disposition mechanisms to enhance the intestinal absorption of etoposide, while preventing its efflux and metabolic inactivation during its transit from intestine to the systemic circulation. A similar effect was observed with curcumin which significantly increased the bioavailability of oral etoposide, while the pharmacokinetics of etoposide after intravenous application was not affected. Therefore, the enhanced oral bioavailability of etoposide in the presence of curcum might be due to inhibition of ABCB1 in the small intestine and possibly due to reduced first-pass metabolism via CYP3A also in the small intestine.^84^

Known interactions of oral etoposide with various drugs including their quantitative effects are summarized in Table 1. To our knowledge, interactions with other drugs are not well documented; however, this does not necessarily mean no interactions exist.

Some other drugs, like ifosfamide^34^, phenytoin and phenobarbitone^85^, also modify systemic exposure (reduced AUC) of etoposide when administered concomitantly. However, in all these cases etoposide was administered intravenously.

On the other hand, low and variable etoposide bioavailability may be related also to its poor solubility in water and chemical instability in physiological fluids. Etoposide’s aqueous solubility is considered as extremely low. The mean solubility of etoposide at 37°C over the pH range 1.30 to 10 is 116.44 to 167.25 μg/ml, respectively.^86^ Assuming that stomach and intestine contain approximately 250 ml of fluid, the initial amount of solute in upper gastrointestinal tract is approximately 30 to 40 mg. Therefore, solubility may play an important role in higher doses.^39^ Extensive degradation of etoposide is observed at pH 1.30 and 10. The intrinsic dissolution rate of etoposide increase with temperature, however, its magnitude is far less than 1.0 mg/min/cm^2^ at 37°C, *i.e.* the absorption is limited by the dissolution rate. Additional proof for etoposide absorption to be dissolution rate limited rather than permeation rate limited is its partition coefficient between n-octanol and water which is 9.94 at 25°C, reflecting etoposide’s high lipophilicity and consequently good permeability. The low aqueous solubility and slow intrinsic dissolution rate may account for the low and variable bioavailability of the drug. However, the problem of poor drug dissolution rate was resolved by the development of hydrophilic preparation: a soft gelatine capsule containing etoposide in the form of solution.^87^ Additional factors that could contribute to the low and erratic bioavailability of etoposide is its chemical instability in physiological (gastric and intestinal) solutions. It is known that pH of the gastrointestinal tract ranges from 1 to 8. Considering etoposide’s pH stability range, its maximal stability is at pH of 5–6.15, while it rapidly degrades at pH<2.03 and pH>8.^30^,^86^*In vitro* studies showed that the decrease in stability in intestinal fluid at pH 7.5 is concentration-dependent while there is no concentration effect on stability in gastric fluid at pH 3.0.^88^

## Safety and efficacy of oral versus intravenous etoposide in SCLC

Safety and efficacy were shown to correlate with AUC of etoposide. Oral administration may increase the variability in AUC and may lead to a greater variability in safety and efficacy of oral etoposide.^30^

The first randomized phase II study compared 3-day oral *vs*. intravenous etoposide schedule in combination with cisplatin in SCLC patients, and assuming 50% bioavailability. Results of this study showed that overall response rates (complete and partial response), time to progression and survival were comparable for both treatment arms. Overall toxicity for both treatment arms was similar and included neutropenia, thrombocytopenia, anemia, alopecia, nausea, diarrhea, vomiting and weight loss. Septic episodes in neutropenic patients as well as moderate to severe anemia and more than 10% weight loss occurred more frequently with the intravenous when compared with the oral treatment. Based on this data it was concluded that the oral treatment regimen could be a suitable substitute for those patients to whom parenteral therapy cannot be given.^24^

Comparable results in terms of response were obtained in another SCLC study which compared safety and efficacy of intravenous and oral etoposide alone, in a 5-day schedule and not assuming 50% bioavailability. Intravenous dose was 80 mg/m^2^/day while oral dose was 130 mg/m^2^/day. Each study gave a similar response rate. The major dose-limiting factor, leukopenia, was observed more frequently in the intravenous administration. Other side effects were anemia, thrombocytopenia, anorexia, nausea, and alopecia^89^,^90^

Yet, two other randomized studies compared oral and intravenous etoposide administration in combination with cisplatin for the treatment of SCLC patients. In both studies etoposide was administered intravenously for 3 days and oral etoposide was administered for 21 days. Self-evidently, the daily dose of intravenously administered etoposide was higher than the dose of orally administered one, while the cumulative dose of etoposide per cycle was higher for orally administered etoposide. Results of both studies showed that the two schedules of etoposide in combination with cisplatin did not result in significant differences in treatment outcome with respect to tumor response and survival. However, a significantly greater rate of hematologic toxicity was noted in intravenous etoposide treatment schedule in the first study^91^ and in oral etoposide treatment schedule in the second one.^92^

As expected, two randomized trials in patients with SCLC demonstrated that oral etoposide alone was inferior to intravenous combination treatment. Of note, although being less effective, oral etoposide alone was associated in the first trial with increased toxicity. However, in both studies intravenous etoposide was used in combined regimens with cisplatin or cyclophosphamide, doxorubicin, and vincristine, while oral etoposide was administered as monochemotherapy. Treatment schedules of oral and intravenous etoposide were also very different.^93^,^94^

Aforementioned clinical trials are described in detail in Table 2.

## Improvement of bioavailability of oral etoposide

### Concomitant medications

Several strategies were explored to improve low and variable bioavailability of oral etoposide. A potential strategy for diminishing variability of oral etoposide is to minimize the sources of variability. Cancer patients are at especially high risk for drug interactions because they commonly receive multiple drugs. In addition, it is estimated that 50% of cancer patients use alternative and herbal medicines, often without their doctor’s knowledge. To diminish these risks, it is important to take an accurate medication history which should be updated at each visit. However, all predictable drug interactions are not always avoidable.^69^

Some drugs were reported to be intentionally used to modulate oral availability of etoposide when administered concomitantly. The increase in bioavailability is a consequence of inhibition of metabolic enzymes and/or efflux transporters. One of these drugs is ketoconazole.^80^ Combined use of etoposide with inhibitors of metabolizing enzymes and/or efflux transporters increases etoposide’s bioavailability. Allen *et al*.^40^ stated that raising the bioavailability closer to 100% might eliminate some variability and allow better control of etoposide exposure. On the contrary, Peng Yong *et al*.^80^ showed that ketoconazole does not reduce the variability.

However, modulation of intestinal absorption of drugs that are substrates of metabolic enzymes and transporters is further complicated by the recognition that polymorphic enzymes and transporters can modulate drug uptake.^95^

### Impact on drug and/or drug delivery system

To maximise bioavailability of oral etoposide, efforts should focus on ensuring rapid drug dissolution in the upper gastrointestinal tract, or delaying drug release to target the upper colon. These suggestions are based on results of directional study of etoposide from rabbit small intestine and colon which showed that secretory permeability was greatest in the ileum, whereas values in the upper small intestine and colon were approximately equal, and represented only 50% of the value in the ileum.^39^

Moreover, Zhang *et al*.^96^ have successfully incorporated etoposide into various modified nano-structured lipid carriers. Pharmacokinetic studies revealed improved relative bioavailability (more than 3.5-fold) of etoposide nanostructured lipid carriers to etoposide suspension in rats after oral administration. They elucidated that the enhanced bioavailability by the modified nanostructured lipid carrier formulation might be attributed to uptake of nanoparticles through the GI tract, increased permeability by surfactants, and decreased degradation and clearance.^96^ Furthermore, Wu *et al*.^97^ developed a phospholipid complex self-emulsifying drug delivery system. Compared with etoposide suspension, the relative bioavailability of this formulation after oral administration in rats was enhanced by 60.21-fold.^97^ Zhang *et al*.^98^ used natural solubilizer rubusoside to form etoposide-rubusoside nanoparticles. This method showed a better solubilization effect and capability of improving physical and chemical stability profiles than a softgel capsule containing etoposide in a vehicle consisting of citric acid, glycerin, purified water, and polyethylene glycol 400. This may improve bioavailability and clinical efficacy as well as improve safety, benefiting from the GRAS (generally regarded as safe) status of rubusoside.^98^ On another point, Mo *et al*.^99^ suggested N-octyl-O-sulfate chitosan to be used as a formulation excipient for etoposide, since it has a potential by inhibiting ABCB1 to improve the absorption of etoposide.

Etoposide phosphate, a more water-soluble prodrug of etoposide has also been suggested for oral administration in an attempt to increase bioavailability and reduce inter-individual variability. Chabot *et al*.^100^ reported a 19% higher extent of absorption for etoposide phosphate compared with literature data for oral etoposide while Sessa *et al*.^101^ reported comparable or only slightly better bioavailability of etoposide phosphate compared with oral etoposide. de Jong *et al*. 102 found a small significant increase in bioavailability but inter-individual variability of bioavailability appeared to be unaltered.

### Influence on the rate of gastric emptying

Joel *et al*.^27^ investigated the use of agents that may influence etoposide stability in gastrointestinal tract and, thereby, bioavailability. Results showed that drugs that influence the rate of gastric emptying (metoclopramide, propantheline), improve the stability of etoposide in artificial intestinal fluid (ethanol, bile salts), and that drugs that decrease stomach acidity (cimetidine) had no significant effect on improving the etoposide AUC.^27^

### Individualization of etoposide dosage

Currently, the dose of etoposide is adjusted according to the body-surface area of the individual patient, but this does not yield the desired minimization in individual variation in the pharmacokinetics in adults.^103^

Etoposide is a suitable drug for pharmacokinetically guided dosing, because of its marked inter-individual pharmacokinetic variability, but relatively little intra-patient variation.^104^ Various studies have been performed with dose adjustments based on pharmacokinetic sampling. These studies have involved the administration of etoposide orally or intravenously to treat patients with different kinds of cancer.

Optimisation of oral etoposide dosage was investigated by El-Yazigi *et al.*^105^ in elderly patients with non-Hodgkin’s lymphoma using individual fraction of dose absorbed and the therapeutic drug monitoring (TDM) approach. The extent of absorption (F) was calculated from the AUC generated from first oral and intravenous doses in the same patient. Etoposide was than given orally at a daily dose equivalent to D_oral_/F. The data obtained indicated that adjustment of the oral dose of etoposide in specific group of patients using individual bioavailability data and TDM approach yielded good safety and efficacy results while keeping the toxicity at the level that is similar to that of the intravenous administration.^105^

AUC is the best pharmacokinetic parameter for predicting anticancer pharmacodynamic effects. Precise estimation of AUC based on plasma concentration requires the handling of many blood samples, usually 8–12, which is expensive, time-consuming and inconvenient.^12^,^106^ Several limited sampling models (LSM) that are based only on a few sparse determinations of plasma concentrations and can obtain a good estimate of the AUC for oral etoposide, were developed and validated.^107^–^109^ Several such models were developed also for intravenous etoposide.^106^,^110^–^112^ The use of LSM in targeted dosing study was used in oral etoposide by Ando *et al*.^113^ and in intravenous etoposide by Lowis *et al*.^114^ Ando *et al*.^113^ reported that during the first 4 days of chemotherapy, one 25-mg capsule was taken three times daily. On day 5, the number of etoposide capsules was adjusted to the individualized dose, depending on the mean etoposide concentration on days 3 and 4, to achieve target concentration range of 1.0 to 1.5 μg/ml.^113^

Another approach to optimizing etoposide dosing is to use population pharmacokinetics, which quantify pharmacokinetic variability among individuals who are the target population, and tries to explain the sources of variability. Individual pharmacokinetic parameters are estimated using the Bayesian approach by combining the population pharmacokinetic model with a limited number of plasma drug concentration measurments.^12^,^115^

A population pharmacokinetics of oral etoposide was studied in patients with various tumor types by Nguyen *et al*.^116^ and Toffoli *et al*.^117^ They indicated that the renal function is the most important variable to be taken into account in etoposide dosing.^116^,^117^

Ciccolini *et al*.^118^ presented a Bayesian method for performing dose adjustment of etoposide when administered intravenously. A Bayesian method was proven to efficiently adjust the experimental values to the target values, thus suggesting that this approach could be routinely used for therapeutic drug monitoring of etoposide.^118^

Functional polymorphisms in metabolizing enzymes and ABC transporters are another relevant factors that have to be considered in personalized medicine. The determination of functional polymorphisms in individual patient enables the use of genotype-based dose administration, to ensure minimal adverse drug reactions and maximal therapeutic efficacy.^37^,^119^

On the other hand, pharmacodynamic model was developed and tested for TDM of 21-day oral etoposide in non-small cell lung cancer patients. The model was developed to predict the value of the neutrophil nadir as a function of the etoposide concentration. Depending of the target nadir (grade 3 neutropenia), the dose was adjusted. However, the pharmacodynamic model yields statistically significant results only when considering the population of patients. Conversely, when applied to individual patients for TDM, the model lacks accuracy and precision.^120^,^121^

## Conclusions

Generally, oral etoposide administration compared to intraveneous administration may result in an improvement of patient’s quality of life and reduced costs. Several studies confirmed comparable safety and efficacy of oral and intravenous etoposide. However, a greater use of oral etoposide is limited by its incomplete and variable bioavailability. Many researchers studied various factors that may influence etoposide bioavailability and, attempted to tailor etoposide dose to the individual patient. The strategy of limited sampling and estimation of individual pharmacokinetic parameters by the Bayesian method seems to efficiently adjust experimental values to the target value. Furthermore, dosage adjustment based on pharmacogenetic analysis may be of great importance for individualized treatment of cancer patients in future. Therefore, further studies are needed to show the accuracy and precision of Bayesian method and pharmacogenetic analysis in dosage adjustment of oral etoposide in SCLC patients.

## Figures and Tables

**TABLE 1.** Quantitative effects of etoposide interactions with various drugs that can potentially affect etoposide bioavailability

**Drug**	**Quantitative effect (method)**	**Reference**
Cisplatin or carboplatin	Increased AUC of etoposide (8% with carboplatin, 28% with cisplatin) (patients, in vitro methods)	Thomas *et al.*^70^
Cyclosporine A	Mean increase of AUC of etoposide 89% (patients)	Bisogno *et al*.^71^
Hydroxyzine	Transport of etoposide increased from the luminal site to the serosal site in the jejunum by 2-fold (reduced efflux) (everted rat gut sacks)	Kan *et al.*^72^
Quinidine	Increased serum concentration of oral etoposide more than 2-fold (everted gut sacks prepared from rat jejunum and ileum)	Leu *et al.*^73^
20(S)-Ginsenoside Rh2	AUC of intragastric administration of etoposide in rats increased by 4.52-fold; c_max_ increased by 2.54-fold (rats)	Zhang *et al.*^74^
GF120918	Increased plasma levels of etoposide after oral administration 4–5-fold (wild-type mice)	Allen *et al.*^40^
Kaempferol	The absolute bioavailability of oral etoposide increased by 11.0–12.3%; the relative bioavailability of oral etoposide increased 1.15–1.64-fold; significantly increased c_max_ (rats)	Li *et al.*^75^
Morin	Increased absolute bioavailability of oral etoposide by 35,9% (rats)	Li *et al*.^76^
Quercetin	Increased absolute bioavailability of oral etoposide to 12.7 (quercetin 5 mg/kg) or 13.6% (quercetin 15 mg/kg) (rats)	Li *et al.*^77^
Verapamil	Increased absolute bioavailability of oral etoposide by 1.38 to 1.47-fold (rats)	Piao *et al.*^78^
PSC833 (valspodar)	Increased plasma concentration of orally administered etoposide at least 10-fold (rats)	Keller *et al.*^79^
Ketoconazole	Increased AUC of oral etoposide by a median of 20% (patients)	Peng Yong *et al.*^80^
Food (standard breakfast: milk, cornflakes, sugar, egg, sausage, bread, margarine, orange marmalade and coffee or tea, sweetened to taste)	Decreased AUC of oral etoposide from 40.8±10.7 μgml^−1^h1.7m^−2^ to 35.8±9.8 μgml^−1^h1.7m^−2^ (patients)	Harvey *et al.*^81^
Grapefruit juice	Decreased AUC of oral etoposide of 26.2%; median absolute bioavailability of 50 mg oral etoposide with and without pretreatment with grapefruit juice was 52.4% and 73.2%, respectively (patients)	Reif *et al.*^82^
Piperine analogue	Increased absolute bioavailability of oral etoposide 2.32-fold (in vitro and animal-derived models)	Najar *et al.*^83^
Curcumin	Increased AUC of oral etoposide by 35.1% (curcumin 2 mg/kg) and 50.8% (curcumin 8 mg/kg); increased F of oral etoposide by 36.0% (curcumin 2 mg/kg) and 52.0% (curcumin 8 mg/kg) (rats)	Lee *et al.*^84^

**TABLE 2.** Clinical trials evaluating safety and efficacy of oral *vs*. intravenous (i.v.) etoposide regimen in SCLC

**Trial(ref.)**	**Sample size**	**Treatment regimen**	**Results (oral etoposide regimen vs. i.v. etoposide regimen)**			
			**ORR (%)**	**mPFS (months)**	**mOS (months)**	**toxicity**
Randomized phase II^24^	83 patients	*i.v. etoposide regimen (41 patients):* cisplatin 100 mg/m^2^ i.v. day 1, etoposide 120 mg/m^2^ i.v. day 1–3	50 vs. 59	5.9 *vs*. 6.6	8.6 for either treatment arm	hematologic toxicity comparable in both treatment arms, infectious episodes, moderate to severe anemia and weight loss more predominant with the i.v regimen
		*oral etoposide regimen (42 patients):* cisplatin 100 mg/m^2^ i.v. day 1, etoposide 120 mg/m^2^ i.v. day 1 and 240 mg/m^2^ orally day 2 and 3				
		Every 4 weeks, maximum of 6 cycles.				
Randomized phase II^89^,^90^	47 patients	*i.v. etoposide regimen (22 patients):* etoposide 80 mg/m^2^ i.v. 5 consecutive days	similar for either treatment arm (PR: 28 vs. 36.4)	**/**	**/**	leukopenia observed in 32% patients of the oral administration and in 59% patients of the i.v. administration
		*oral etoposide regimen (25 patients):* etoposide 130 mg/m^2^ orally 5 consecutive days				
Randomized trial^91^	21 patients	*i.v. etoposide regimen (14 patients):* cisplatin 80 mg/m^2^ i.v. day 1, etoposide 100 mg/m^2^ i.v. day 2, 3 and 4	86 *vs*. 64	**/**	no significant difference	hematologic toxicity less severe for oral regimen than for i.v. regimen
		*oral etoposide regimen (7 patients):* cisplatin 80 mg/m^2^ i.v. day 1, etoposide 50 mg orally day 3–23				
		Both regimens were repeated every 4 weeks.				
Randomized phase III^92^	306	*i.v. etoposide regimen:* cisplatin 25 mg/m^2^ i.v. 3 days, etoposide 130 mg/m^2^ i.v. 3 days Regimen was repeated every 21 days for 8 cycles.	14 *vs*. 15 (PR: 47 *vs*. 42)	7 months for either treatment arm	9.9 *vs*. 9.5	lethal toxicity due to neutropenia and infection: in 10% of patients on oral etoposide regimen and in 4% on i.v. etoposide regimen (difference not statistically significant)
		*oral etoposide regimen:* cisplatin 33 mg/m^2^ i.v. 3 days, etoposide 50 mg/m^2^ orally 21 days Regimen was repeated every 28 days for 6 cycles.				
Randomized trial^93^	339 patients	*i.v. etoposide regimen (168 patients):* standard intravenous regimen of etoposide and vincristine, or cyclophosphamide, doxorubicin, and vincristine, 4 cycles	45 *vs*. 51	**/**	4.3 *vs*. 6.1	grade 2 or worse haematological toxicity: in 29% of patients on oral etoposide regimen and in 21% on i.v. etoposide regimen
		*oral etoposide regimen (171 patients):* etoposide 50 mg orally twice daily for 10 days, 4 cycles				
Randomized trial^94^	155 patients	*i.v. etoposide regimen (80 patients):* intravenous regimen consisting of alternating cycles of etoposide and cisplatin and cyclophosphamide, doxorubicin, and vincristine	32.9 *vs*. 46.3	3.6 *vs.* 5.6	4.8 *vs*. 5.9	toxicity similar in the two treatment arms
		*oral etoposide regimen (75 patients):* etoposide 100 mg orally twice daily for 5 days				
		Both regimens were repeated every 21 days for 6 cycles.				

ORR = overall response rate; mPFS = median progression free survival; mOS = median overall survival; PR = partial respons
